# In Vitro and In Vivo Anti-Breast Cancer Activities of Some Newly Synthesized 5-(thiophen-2-yl)thieno-[2,3-d]pyrimidin-4-one Candidates

**DOI:** 10.3390/molecules24122255

**Published:** 2019-06-17

**Authors:** Abd El-Galil E. Amr, Alhussein A. Ibrahimd, Mohamed F. El-Shehry, Hanaa M. Hosni, Ahmed A. Fayed, Elsayed A. Elsayed

**Affiliations:** 1 Pharmaceutical Chemistry Department, Drug Exploration & Development Chair (DEDC), College of Pharmacy, King Saud University, Riyadh 11451, Saudi Arabia; 2 Applied Organic Chemistry Department, National Research Center, Cairo, Dokki 12622, Egypt; alhusseina62@yahoo.com (A.A.I.); dr_ahmedfayed14@yahoo.com (A.A.F.); 3 Pesticide Chemistry Department, National Research Center, Dokki 12622, Cairo, Egypt; hanaanrc@yahoo.com (M.F.E.-S.); hanaahosni434@yahoo.com (H.M.H.); 4 Chemistry Department, Al-Zahrawy University College, Karbala 56001, Iraq; 5 Respiratory Therapy Department, College of Medical Rehabilitation Sciences, Taibah University, Madinah Munawara, 22624, Saudi Arabia; 6 Zoology Department, Bioproducts Research Chair, Faculty of Science, King Saud University, Riyadh 11451, Saudi Arabia; eaelsayed@ksu.edu.sa; 7 Chemistry of Natural and Microbial Products Department, National Research Centre, Dokki 12622, Cairo, Egypt

**Keywords:** thiopene, thienopyrimidinone, thiazolidinone, anticancer activity

## Abstract

In this study, some of new thiophenyl thienopyrimidinone derivatives **2**–**15** were prepared and tested as anti-cancer agents by using thiophenyl thieno[2,3-d]pyrimidinone derivative **2** as a starting material, which was prepared from cyclization of ethyl ester derivative **1** with formamide. Treatment of **2** with ethyl- chloroacetate gave thienopyrimidinone *N*-ethylacetate **3**, which was reacted with hydrazine hydrate or anthranilic acid to afford acetohydrazide **4** and benzo[d][1,3]oxazin-4-one **5,** respectively. Condensation of **4** with aromatic aldehydes or phenylisothiocyanate yielded Schiff base derivatives **6,7**, and thiosemicarbazise **10**, which were treated with 2-mercaptoacetic acid or chloroacetic acid to give the corresponding thiazolidinones **8**, **9,** and phenylimino-thiazolidinone **11**, respectively. Treatment of **4** with ethylacetoacetate or acetic acid/acetic anhydride gave pyrazole **12** and acetyl acetohydrazide **13** derivatives, respectively. The latter compound **13** was reacted with ethyl cycno-acetate or malononitrile to give **14** and **15**, respectively. In this work, we have studied the anti-cancer activity of the synthesized thienopyrimidinone derivatives against MCF-7 and MCF-10A cancer cells. Furthermore, in vivo experiments showed that the synthesized compounds significantly reduced tumor growth up to the 8^th^ day of treatment in comparison to control animal models. Additionally, the synthesized derivatives showed potential inhibitory effects against pim-1 kinase activities.

## 1. Introduction

Cancer is a major health problem acting as a global killer, so synthesizing new compounds, which may act as potent antitumor agents, is a great target for chemists working in this field. In this study, we are interested in synthesizing and studying biological activities of thieno[2,3-d]pyrimidinone derivatives [1,2,3,4,5,6,7,8,9,10,11]. Thienopyrimidinones are very important moieties that act as keys for pharmacological and pharmaceutical properties. They are reported to cause antiviral [12], antimicrobial [13], antihypertensive [14], analgesic, and anti-inflammatory activities [15]. They also inhibit various protein kinase enzymes, such as CK2 involved in particular anticancer activity [16]. Additionally, the nitrogenous ring system was associated with some types of biological activities such as: anti-inflammatory [17], insecticidal [18], antimicrobial and antituberculosis [19,20] activities. On the other hand, thienopyrimidinones contain a thiophene ring fused with a pyrimidinone nucleus. In general, this system was thought to be interesting in development of pharmaceutical compounds [21,22], and was not only evaluated as cGMP phosphodiesterase inhibitors [23], anti-viral [24], anti-inflammatory [25], anti-microbial agents [26], but also as kinase inhibitors and potential anti-cancer agents [27,28]. In continuation to our previous work, and to extend our research [1,2,3,4,5,6,7,8,9,10,11], from the above points, we have studied the anticancer activity of the newly synthesized substituted thienopyrimidinone derivatives against MCF-7 and MCF-10A cancer cells. Furthermore, the work was extended to evaluate the effects of synthesized derivatives on the inhibition of tumor growth in an in vivo animal model. Finally, we evaluated the inhibitory effects of our synthesized compounds against pim-1 kinase activity as a possible mechanism of their action.

## 2. Results and Discussion

### 2.1. Chemistry

**Scheme 1:**
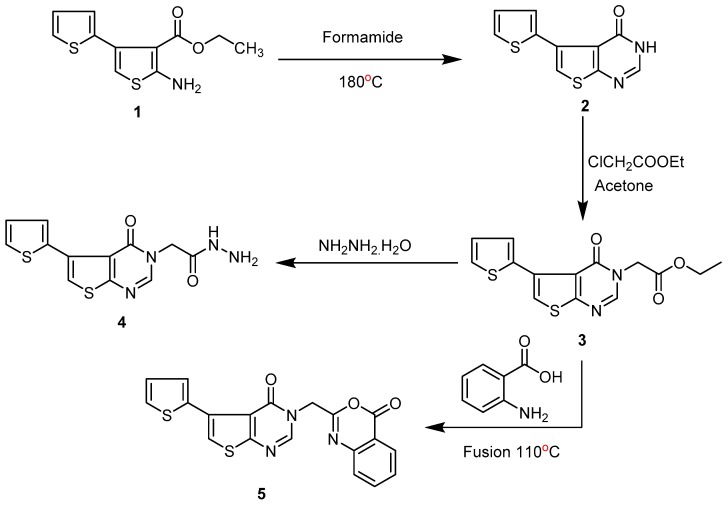
Synthetic route for compounds **2**–**5**.

A series of thiophenyl thienopyrimidinone derivatives **2**–**15** were prepared and tested as anti-cancer agents. Cyclization of ethyl 5’-amino-[2,3’-bithiophene]-4’-carboxylate (**1**) with formamide gave the corresponding thiophenylthieno[2,3-d]pyrimidinone derivative (**2**), which was treated with ethylchloroacetate to give thienopyrimidinone N-ethylacetate **3**. Reaction of **3** with hydrazine hydrate or anthranilic acid afforded the corresponding hydrazide **4** and benzooxazinone **5** derivatives, respectively (Scheme 1).

**Scheme 2:**
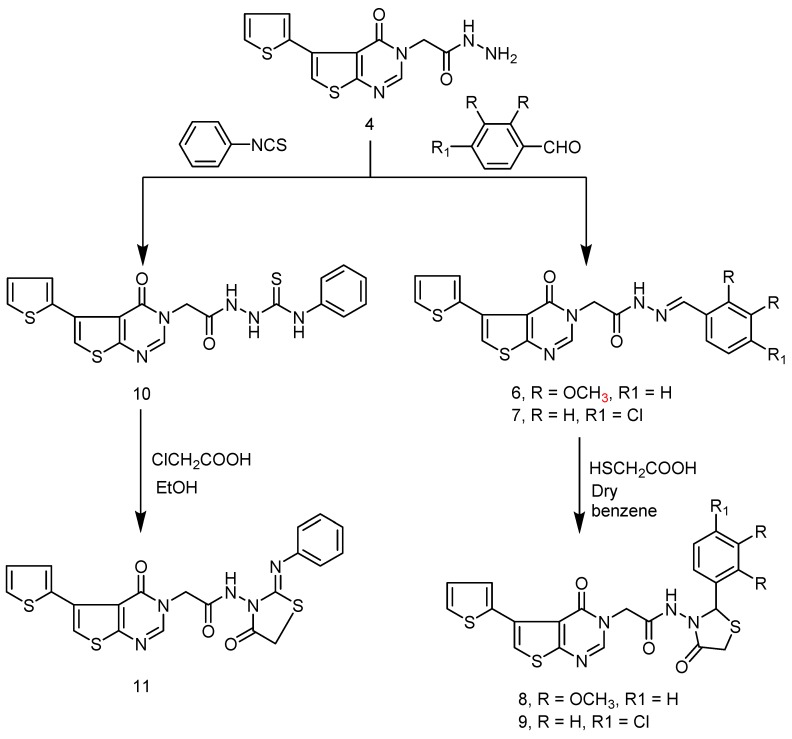
Synthetic route for compounds **6**–**11**.

Condensation of **4** with aromatic aldehydes, namely, 2,3-dimethoxybenzaldehyde or 4-chlorobenzaldehyde gave the corresponding Schiff base derivatives **6** and **7**, which were cyclized via reaction with 2-mercaptoacetic acid in dry benzene to give the corresponding thiazolidinone derivatives **8** and **9**, respectively. Treatment of **4** with phenylisothiocyanate gave thiosemicarbazide **10**, which was condensed with chloroacetic acid to afford phenyliminothiazolidinone derivative **11** (Scheme 2).

**Scheme 3:**
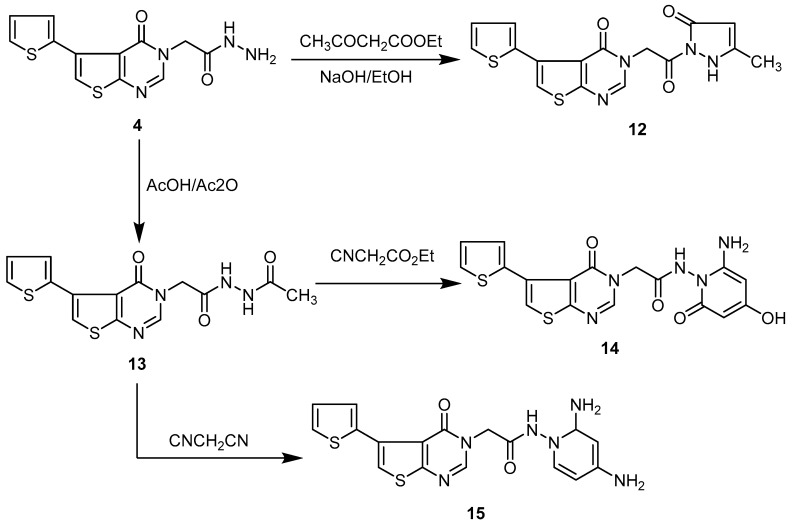
Synthetic route for compounds **12**–**15**.

Finally, treatment of **4** with ethylacetoacetate or acetic acid/acetic anhydride gave the corresponding pyrazolyl derivative **12** and N-acetyl hydrazide **13**, respectively. The latter compound **13** was reacted with ethylcycnoacetate or malononitrile to give pyridine derivatives **14** and **15**, respectively (Scheme 3).

### 2.2. Biological Evaluation

**Figure 1:**
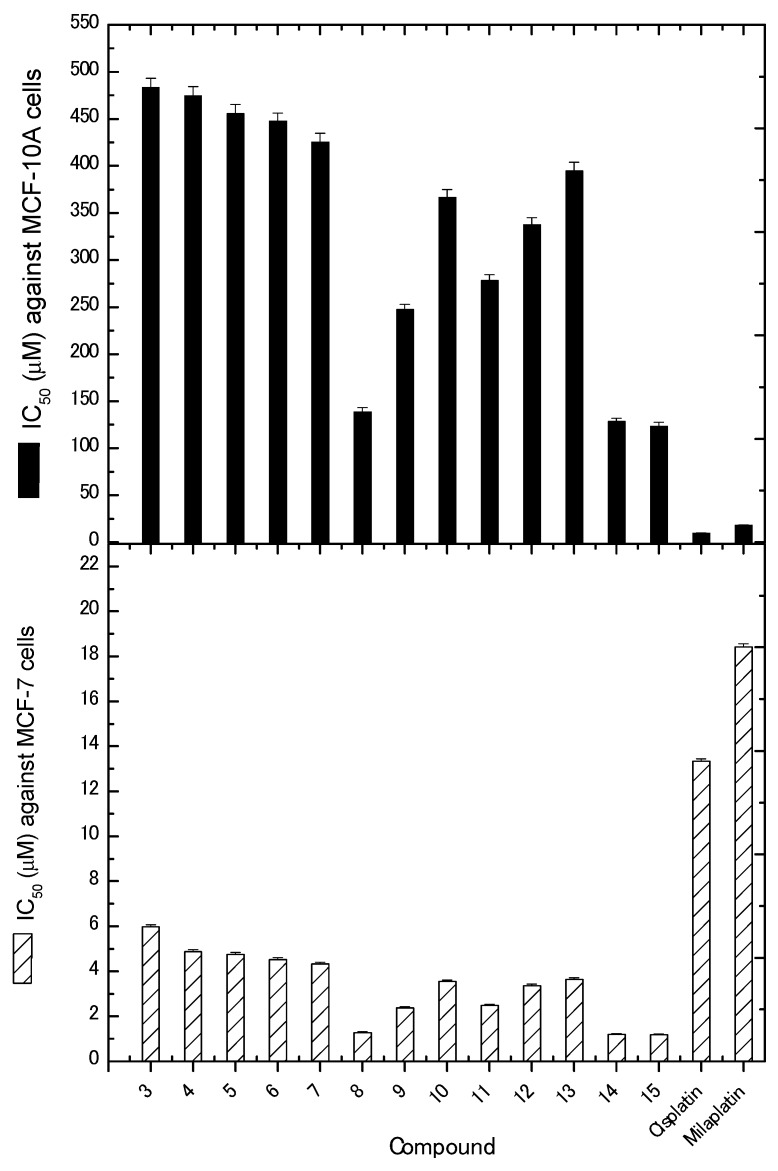
Obtained IC_50_ values for MCF-7 and MCF-10A cells.

MCF-7 cells were used to investigate the potential in vitro anti-proliferative potential of the synthesized compounds. With the exception of Cpd. **2** (data not shown), we found that all compounds have promising activities when used in µM concentration. On the other hand, Cisplatin and Milaplatin showed higher IC_50_ values (13.34 ± 0.11 and 18.43 ± 0.13 µM, respectively). DMSO at concentrations of 0.1% and 0.5%, had little or no toxicity, whereas higher concentrations inhibited the growth of MCF-7cells. Therefore, it seems DMSO could be solvents of choice acceptable to be used at concentrations < 0.5% (*v*/*v*) towards the examined cells and possibly for other cell lines. Also, the effect on cell viability was proportional to the concentration applied. From Figure 1, we can see that Cpd. **15, 14** and **8** (IC_50_, 1.18 ± 0.032, 1.19 ± 0.042, 1.26 ± 0.052 µM, respectively) followed by **9** and **11** (IC_50_, 2.37 ± 0.053 and 2.48 ± 0.054 µM respectively) produced the highest effect on cell viability. Secondly, compounds **12**, **10** and **13**, showed moderate activities (IC_50_, 3.36 ± 0.063, 3.55 ± 0.065 and 3.64 ± 0.074 µM, respectively). Compounds **7, 6, 5, 4** and **3** were the least active ones (IC_50_, 4.33 ± 0.076, 4.52 ± 0.085, 4.76 ± 0.087, 4.87 ± 0.098 and 5.98 ± 0.099 µM, respectively). The order of activities can be arranged as **15** > **14** > **8** > **9 > 11** > **12** > **10** > **13** > **7** > **6** > **5** > **4** > **3**. 

Results revealed that the substitution with pyridine moiety at the terminal NH improved the cytotoxic effect than the pyrimidone derivatives. In contrast, substitution with 5-membered di-heterocyclic ring system with aryl moiety decreased the obtained activities (methoxy phenyl > chlorophenyl). Attaching five membered pyrazolinone ring system bearing no aryl moiety at terminal NH (compound **12** decreased the activities than those containing aryl substitutions (compounds **9** and **11**). Compounds **10, 7** and **6** that contain aromatic *N*-substitution still have more potent activity. The increased effect of the aromatic ring may be attributed to ring aromaticity and electron resonance. On the other hand, aliphatic side chains (compounds **4** and **3**) or methylene bridges (Compound **5**) have less potent activities.

Additionally, results against non-tumorigenic MCF-10A proved that our derivatives have higher degrees of safety towards normal cells.

#### In Vivo Xenograft Model 

**Figure 2:**
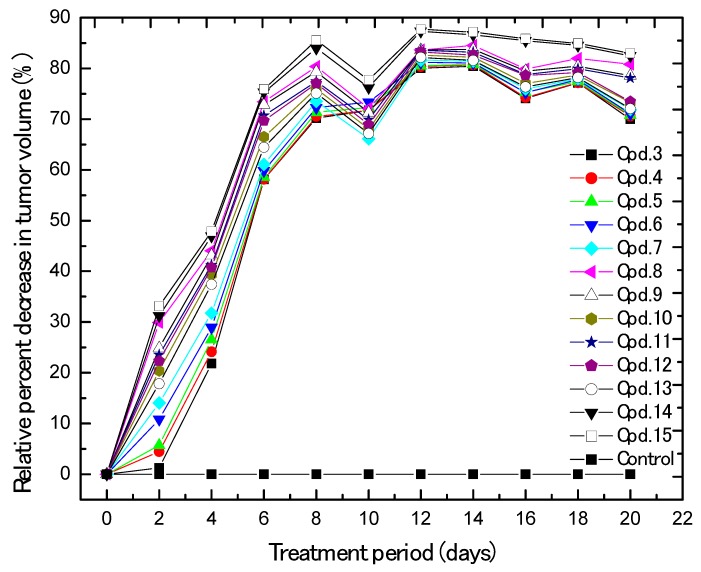
Relative percentage of decrease in tumor volume in response to prepared compounds.

The in vivo anti-breast cancer activities of different synthesized derivatives were evaluated using a breast cancer mouse xenograft model. Figure 2 shows the increase in percentage of inhibition in tumor growth with treatment time when animals were exposed to different compounds. This was also compared with tumor development in control animals. It can be seen, that our derivatives reduced tumor growth starting day 2. The maximal effect was obtained after 8 days. Furthermore, the in vivo effect showed also the same inhibitory pattern obtained in the in vitro experiments. The average weight of each group of mice treated with drug and the control group summarized in Table 1.

**Figure 3:**
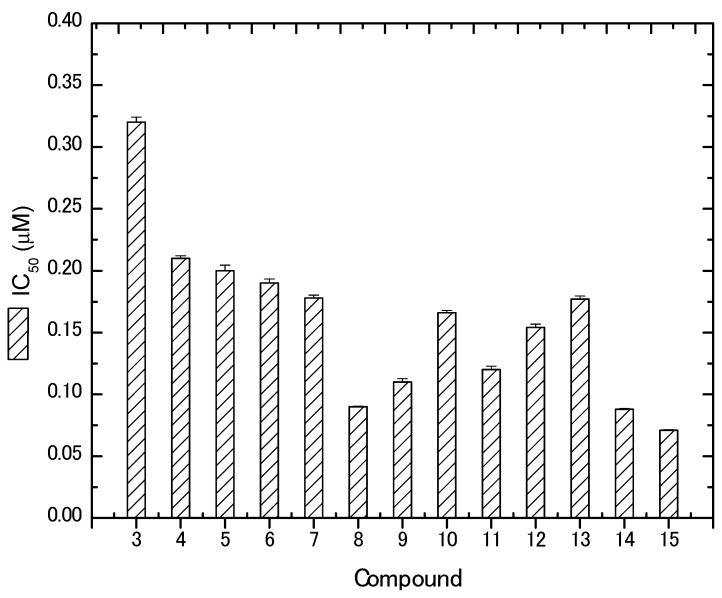
IC_50_ of the tested compounds against pim-1 Kinase.

The Provirus Integration in Maloney (Pim) kinases represents a family of constitutively active serine/threonine kinases and includes three subtypes (pim-1, pim-2 and pim-3). Pim kinases regulate many biological processes such as cell cycle, cell proliferation, apoptosis and drug resistance [29,30,31,32]. Being expressed in many types of solid and hematological cancers and almost absent in benign lesions, pim kinases proved to be a successful anti-cancer drug target of low toxicity [33,34,35,36,37,38,39,40]. Results obtained in Figure 3 showed that all synthesized compounds were showed potent inhibitory effects against pim-1 kinase.

## 3. Materials and Methods 

### 3.1. Chemistry

“Melting points were determined in open glass capillary tubes with an Electro Thermal Digital melting point apparatus (model: IA9100) and are uncorrected. Elemental microanalyses were carried out in the microanalysis unit of NRC and were found within the acceptable limits of the calculated values. Infrared spectra (KBr) were recorded on a Nexus 670 FTIR Nicolet, Fourier Transform infrared spectrometer. ^1^H- and ^13^C NMR spectra were run in (DMSO-d_6_) on Jeol 500 MHz instruments. Mass spectra were run on a MAT Finnigan SSQ 7000 spectrometer, using the electron impact technique (EI).”.

*Synthesis of 5-(thiophen-2-yl)thieno[2,3-d]pyrimidin-4(3H)-one **(2).*** A mixture of compound **1** (1 mmol, 253 mg) and formamide (20 mL) was heated at 180 °C in oil bath for 2 h. The formed solid was collected by filtration, washed with cold methanol, dried and crystallized from EtOH to give compound **2**. Yield 80%, M.p 192–194 °C; IR (KBr, cm^−1^): ῡ 3323 (NH), 1659 (C=O). ^1^H NMR (DMSO-d_6_) δ_H_: 7.11–7.72 (m, 4H, thiophene-H), 8.50 (s, 1H, CH-pyrimidine), 13.30 (s, 1H, NH, disappeared with D_2_O). ^13^C NMR: 119.98, 122.01, 122.17, 126.84, 127.75, 128.74, 131.26, 136.42, (8C, thiophene-C), 157.05 (1C, pyrimidine-C), 165.56 (C=O). Mass spectrum, *m*/*z* (EI, %): 234 (M^+^, 100), 235 (M^+^ + 1, 11), 236 (M^+^ + 2, 9). Analysis for C_10_H_6_N_2_OS_2_ (234.29): Calculated: C, 51.27; H, 2.58; N, 11.96; S, 27.37. Found: C, 51.20; H, 2.50; N, 11.90; S, 27.30.

*Synthesis of ethyl 2-(4-oxo-5-(thiophen-2-yl)thieno[2,3-d]pyrimidin-3(4H)-yl)acetate **(3).*** A mixture of **2** (1 mmol, 234 mg), ethylchloroacetate (1 mmol, 122 mg) and anhydrous potassium carbonate (8 mmol) in dry acetone (30 mL) was heated under reflux for 4h. The obtained solid was removed by filtration, the filtrate was concentrated, the precipitate solid was filtered off, dried, and crystallized from EtOH to give the ester derivative **3**. Yield 70%, m.p 135–137 °C. IR (KBr, cm^−1^): ν 1753 (C=O, ester), 1655 (CO); ^1^H NMR (DMSO-d_6_), δ: 1.24 (t, 3H, CH_3_), 4.20 (s, 2H, CH_2_), 4.85 (q, 2H, CH_2_-ethyl), 7.10–7.72 (m, 4H, thiophene-H), 8.50 (s, 1H, pyrimidine-H). ^13^C-NMR (DMSO)-d_6_) δc: 14.5 (CH_3_), 40.2 (CH_2_), 47.8 (CH_2_), 119.9, 122.2, 126.9, 127.8, 128.7, 128.9, 131.3, 136.4 (8C, thiophene-C), 157.0 (1C, pyrimidine-C), 165.6, 168.3 (2C, 2CO). Mass spectrum, *m*/*z* (EI, %): 320 (M^+^, 100), 321 (M^+^ + 1, 18). Analysis for C_14_H_12_N_2_O_3_S_2_ (320.38): Calculated: C, 52.49; H, 3.78; N, 8.74; S, 20.01. Found: C, 52.40; H, 3.70; N, 8.68; S, 19.86.

*Synthesis of 2-(4-oxo-5-(thiophen-2-yl)thieno[2,3-d]pyrimidin-3(4H)-yl)acetohydrazide **(4).*** To a solution of **3** (1 mmol, 320 mg) in ethanol (50 mL), hydrazine hydrate (4 mmol, 85%) was added and refluxed for 8 h. The precipitated solid was collected by filteration, dried and crystallized from EtOH to give compound **4**. Yield 75%, m.p. 205–207 °C, IR (KBr, cm^−1^): ν 3322 (NH), 3246 (NH_2_), 1659 (C=O). ^1^H-NMR (DMSO-d_6_) δ: 3.39 (s, 2H, CH_2_), 4.65 (s, 2H, NH_2_, disappeared with D_2_O), 7.10–7.65 (m, 4H, thiophene-H), 8.17 (s, 1H, pyrimidine-H), 12.58 (s, 1H, NH, disappeared with D_2_O). ^13^C-NMR (DMSOd_6_) δc: 40.1 (CH_2_), 120.5, 120.8, 126.6, 127.8, 128.7, 131.4, 133.5, 136.8 (8C, thiophene-C), 157.9 (1C, pyrimidine-C), 166.3, 169.4 (2C, 2CO). Mass spectrum, m/z (EI, %): 306 (M^+^, 100), 307 (M^+^ + 1, 14). Analysis for C_12_H_10_N_4_O_2_S_2_ (306.36): Calculated: C, 47.05; H, 3.29; N, 18.29; S, 20.93. Found: C, 46.85; H, 3.20; N, 18.20; S, 20.85.

*Synthesis of 2-((4-oxo-5-(thiophen-2-yl)thieno[2,3-d]pyrimidin-3(4H)-yl)methyl)-4H-benzo[d]*-[1,3] *oxazin-4-one **(5).*** A mixture of **3** (1 mmol, 320 mg) and anthranilic acid (1 mmol, 137 mg) was fused together at 110 °C in an oil bath for 3hr. The residue was boiled with ethanol, the formed solid was removed by filtration, the solid formed was filtered off, and crystallized from EtOH to give **5**. Yield 60%, m.p. 225–227 °C. IR (KBr, Cm^−1^): ν 1750 (C=O), 1684 (C=O). ^1^H-NMR (DMSO d_6_) δ_H_: 4.58 (s, 2H, CH_2_), 7.10–7.54 (m, 4H, thiophene-H), 7.68-8.16 (m, 4H, Ph-H), 8.64 (s, 1H, pyrimidine-H). ^13^C-MNR (DMSO-d_6_) δ_c_: 43.0 (CH_2_), 120.0, 120.1, 121.3, 121.7, 126.7, 126.8, 127.7, 128.7, 131.3, 136.5, 136.6, 149.5, 149.7, 158.2 (14C, thiophene + Ph-C), 157.2 (1C, pyrimidine-C), 160.1, 165.6, 166.5 (3C, 3C=O). Mass spectrum, *m*/*z* (EI, %): 393 (M^+^, 100). Analysis for C_19_H_11_N_3_O_3_S_2_ (393.44): Calculated: C, 58.00; H, 2.82; N, 10.68; S, 16.30. Found: C, 57.90; H, 2.78; N, 10.60; S, 16.25.

*Synthesis of hydrazone derivatives **6** and **7**.* To a mixture of **4** (1 mmol, 306 mg) and aromatic aldehydes, namely 3,4-dimethoxybenazaldehyed or 4-chlorobenzaldehyde (1 mmol) in ethanol (50 mL), few drops of piperidine were added and refluxed for 5 h, with stirring. After cooling, the formed solid was filtered off and recrystallized from dioxan to give the corresponding derivatives **6** and **7** respectively.

*N′-(2,3-Dimethoxybenzylidene)-2-(4-oxo-5-(thiophen-2-yl)thieno[2,3-d]pyrimidin-3(4H)-yl)-acetohydrazide **(6).*** Yield 68%, m.p. 248–250 °C. IR (KBr, cm^−1^): ν 3388 (NH), 1660 (C=O). ^1^H-NMR (DMSO-d_6_) δ_H_: 3.72, 3.86 (2s, 6H, 2OCH_3_), 4.11 (s, 2H, CH_2_), 6.98-7.61 (m, 7H, thiophene + Ph-H), 8.60 (s, 1H, CH=N), 9.05 (s, 1H, pyrimidine-H), 10.51 (s, 1H, NH, disappeared with D_2_O). ^13^C-NMR (MDSO-d_6_) δc: 44.1 (1C, CH_2_), 56.1, 60.1 (2C, OCH_3_), 114.3, 116.1, 119.5, 121.8, 124.0, 126.7, 127.7, 128.7, 129.3, 130.6, 131.3, 136.47, 149.07, 149.77 (14C, thiophene-C + Ph-C), 148.56 (1C, CH=N), 157.19 (1C, pyrimidine-C), 163.66, 169.74 (2C, 2C=O). Mass spectrum, *m*/*z* (EI, %): 454 (M^+^, 100). Analysis for C_21_H_18_N_4_O_4_S_2_ (454.52): Calculated: C, 55.49; H, 3.99; N, 12.33; S, 14.11. Found: C, 55.40; H, 3.90; N, 12.25; S, 13.96.

*N′-(4-Chlorobenzylidene)-2-(4-oxo-5-(thiophen-2-yl)thieno[2,3-d]pyrimidin-3(4H)-yl)acetohydrazide **(7).*** Yield 65%, m.p. 250–252 °C. IR (KBr, Cm^−1^): ν 3408 (NH), 1670 (CO), 1659 (CO). ^1^H-NMR (DMSO-d_6_) δH: 4.10 (s, 2H, CH_2_), 7.10–7.80 (m, 9H, Ar-H + CH=N), 9.05 (s, 1H, pyrimidine-H), 10.56 (s, 1H, NH, disappeared with D_2_O). ^13^C-NMR (MDSO-d_6_) δc: 44.1 (1C, CH_2_), 145.2 (1C, CH=N), 119.5, 121.9, 124.0, 126.8, 127.8, 128.1, 129.3, 130.6, 131.3, 136.5, 149.1, 149.8 (14C, thiophene-C + Ph-C), 157.5 (1C, pyrimidine-C), 162.7, 169.9 (2C, 2C=O). Mass spectrum, *m*/*z* (EI, %): 428 (M^+^, 100), 430 (M^+^ + 2, 40). Analysis for C_19_H_13_ClN_4_O_2_S_2_ (428.91): Calculated: C, 53.21; H, 3.06; N, 13.06; S, 14.95. Found: C, 53.12; H, 3.00; N, 13.00; S, 14.88.

*Synthesis of thiazolidinone derivatives **8** and **9**.* To a stirred solution of **6** or **7** (1 mmol) in dry benzene (40 mL), thioglycollic acid (1 mmol, 92 mg) in dry benzene (10 mL) was added and refluxed for 12 h. The solvent was evaporated to dryness. The formed product was collected, and crystallized with dioxan to obtain the corresponding products **8** and **9**, respectively.

*N-(2-(2,3-Dimethoxyphenyl)-4-oxothiazolidin-3-yl)-2-(4-oxo-5-(thiophen-2-yl)thieno[2,3-d]pyrimidin-3(4H)-yl)acetamide **(8).*** Yield 60%, m.p. 280–282 °C. IR (KBr, cm^−1^): ν 3417 (NH), 1670, 1680, 1630 (3 C=O). ^1^H-NMR (DMSO-d_6_) δ_H_: 3.65, 3.90 (2s, 6H, 2OCH_3_), 4.66 (s, 2H, CH_2_), 4.77 (s, 2H, CH_2_), 5.86 (s, 1H, CH), 6.95-7.65 (m, 7H, thiophene + Ph-H), 8.49 (s, 1H, pyrimidine-H), 10.84 (s, 1H, NH, disappeared with D_2_O). ^13^C-NMR (DMSO-d_6_) δ_c_: 35.9, 47.4 (2C, 2CH_2_), 56.5, 58.42 (2C, 2OCH_3_), 59.2 (1C, CH), 113.4, 117.9, 118.9, 120.7, 121.8, 126.8, 127.5, 128.7, 130.7, 131.0, 136.5, 145.3, 149.5, 149.9 (14C, thiophene-C + Ph-C), 156.9 (1C, pyrimidine-C), 162.5, 165.4, 169.2 (3C, 3C=O). Mass spectrum, *m*/*z* (EI, %): 528 (M^+^, 100), 529 (M^+^ + 1, 30). Analysis for C_23_H_20_N_4_O_5_S_3_ (528): Calculated: C, 52.26; H, 3.81; N, 10.60; S, 18.19. Found: C, 52.18; H, 3.75; N, 10.52; S, 18.10.

*N-(2-(4-Chlorophenyl)-4-oxothiazolidin-3-yl)-2-(4-oxo-5-(thiophen-2-yl)thieno[2,3-d]-pyrimidin-3(4H)-yl)- acetamide **(9).*** Yield 75%, m.p. 175-177 °C. IR (KBr, cm^−1^): ν 3417 (NH), 1720, 1630, 1660 (3C=O). ^1^H-NMR (DMSO-d_6_) δ_H_: 3.81, 5.16 (2s, 4H, 2CH_2_), 5.90 (s, 1H, CH), 7.08–7.72 (m, 8H, thiophene-H + Ph-H), 8.50 (s, 1H, pyrimidine-H), 10.92 (s, 1H, NH, disappeared with D_2_O). ^13^CNMR (DMSO-d_6_) δc: 40.1, 48.2 (2C, 2CH_2_), 65.2 (1C, CH), 120.0, 121.8, 127.7, 128.7, 129.3, 129.4, 131.3, 133.3, 135.0, 136.5, 143.5, 149.7 (14C, thiophene-C + Ph-C), 157.2 (1C, pyrimidine-C), 163.8, 165.6, 169.9 (3C, 3C=O). Mass spectrum, *m*/*z* (EI, %): 503 (M^+^, 100), 505 (M^+^ + 2, 34). Analysis for C_21_H_15_ClN_4_O_3_S_3_ (503.01): Calculated: C, 50.14; H, 3.01; N, 11.14; S, 19.12. Found: C, 50.02; H, 3.00; N, 11.04; S, 19.06.

*Synthesis of 2-(2-(4-oxo-5-(thiophen-2-yl)thieno[2,3-d]pyrimidin-3(4H)-yl)acetyl)-N-phenylhydrazine-1- carbothioamide **(10).*** A mixture of **4** (1mmol, 306 mg) and phenylisothiocynate (1 mmol, 135 mg) in dry dioxan (50 mL) was refluxed for 6 h. The obtained solid was filtered off, washed with ether, dried and recrystallized from ethanol to give thiosemicarbazide **10**. Yield 60%, m.p. 240–242 °C. IR (KBr, cm^−1^): ν 3414-3323 (NH), 1680, 1660 (2CO). ^1^H-NMR (DMSO-d_6_) δ_H_: 4.66 (s, 2H, CH_2_), 6.95-7.58 (m, 9H, thiophene + Ph-H), 8.49 (s, 1H, pyrimidine-H), 8.70, 10.71, 12.78 (3s, 3H, 3NH, disappeared with D_2_O). ^13^CNMR (DMSO-d_6_) δc: 40.16 (CH_2_), 119.9, 121.8, 126.8, 127.9, 128.8, 129.1, 130.2, 131.3, 134.1, 136.5, 137.9, 149.9 (14C, thiophene + Ph-C), 156.5 (1C, pyrimimidine-C), 166.5, 169.1 (2C, 2C=O), 171.0 (1C, C=S). Mass spectrum, *m*/*z* (EI, %): 441 (M^+^, 100), 442 (M^+^ + 1, 26). Analysis for C_19_H_15_N_5_O_2_S_3_ (441.54): Calculated: C, 51.68; H, 3.42; N, 15.86; S, 21.78. Found: C, 51.60; H, 3.40; N, 15.80; S, 21.70.

*Synthesis of N-(4-oxo-2-(phenylimino)thiazolidin-3-yl)-2-(4-oxo-5-(thiophen-2-yl)thieno[2,3-d]-pyrimidin- 3(4H)-yl)acetamide **(11).*** A mixture of 10 (1 mmol, 441 mg) and chloroacetic acid (1 mmol, 94 mg) in absolute ethanol (30 mL) was heated under reflux for 8 h. The solid formed was filtered off and crystallized with dioxane to give thiazole derivative 11. Yield 60%, m.p. 255–257 °C. IR (KBr, cm^−1^): ν 3420 (NH), 1720, 1630 (2C=O). ^1^H-NMR (DMSO-d_6_) δ_H_: 3.76 (s, 2H, CH_2_), 4.85 (s, 2H, CH_2_), 6.95-7.65 (m, 9H, thiophene-H + Ph-H), 8.50 (s, 1H, pyrimidine-H), 11.10 (s, 1H, NH, disappeared with D_2_O). ^13^CNMR (DMSO-d_6_) δc: 40.2 (CH_2_), 56.8 (CH_2_), 120.0, 122.0, 126.7, 128.7, 130.7, 131.3, 132.5, 136.4, (8C, thiophene-C), 145.2, 149.3, 150.8, 151.9 (6C, Ph-C), 156.6 (1C, pyrimidine-C), 158.1 (1C, C=N), 163.4, 165.7, 169.6 (3C, 3C=O). Mass spectrum, *m*/*z* (EI, %): 481 (M^+^. 100), 482 (M^+^ + 1, 24). Analysis for C_21_H_15_N_5_O_3_S_3_ (481.56): Calculated: C, 52.38; H, 3.14; N, 14.54; S, 19.97. Found: C, 52.30; H, 3.10; N, 14.50; S, 19.90.

*Synthesis of 3-(2-(3-methyl-5-oxo-2,5-dihydro-1H-pyrazol-1-yl)-2-oxoethyl)-5-(thiophen-2-yl)thieno- [2,3-d]pyrimidin-4(3H)-one **(12).*** A mixture of compound 4 (1 mmol, 306 mg) and ethylacetoacetate (1 mmol, 130 mg) in ethanolic sodium hydroxide (0.5 mmol/50 mL) was refluxed with stirring for 6 h. The precipitate was collected by filtration and crystallized from dioxane to give pyrazole derivative 12. Yield 80%, m.p. 225–227 °C. IR (KBr, cm^−1^): ν 3417 (NH), 1650, 1630 (2C=O). ^1^H-NMR (DMSOd_6_) δ_H_: 1.70 (s, 3H, CH_3_), 4.35 (s, 2H, CH_2_), 5.65 (s, 1H, pyrazole-CH), 7.51–7.69 (m, 4H, thiophene-H), 8.46 (s, 1H, pyrimidine-H), 12.93 (s, 1H, NH, disappeared with D_2_O). ^13^C-NMR (DMSO-d_6_) δc: 34.4 (CH_3_), 47.0 (CH_2_), 120.0, 123.8, 127.7, 128.7, 129.4, 130.9, 132.6, 136.5 (8C, thiophene), 98.3, 151.9 (2C, Pyrazole-C), 156.6 (1C, pyrimidine-C), 163.4, 166.5, 169.7 (3C, 3C=O). Mass spectrum, *m*/*z* (EI, %): 372 (M^+^, 100), 373 (M^+^ + 1, 18). Analysis for C_16_H_12_N_4_O_3_S_2_ (372.42): Calculated: C, 51.60; H, 3.25; N, 15.04; S, 17.22. Found: C, 51.50; H, 3.20; N, 15.00; S, 17.16.

*Synthesis of N′-acetyl-2-(4-oxo-5-(thiophen-2-yl)thieno[2,3-d]pyrimidin-3(4H)-yl)aceto-hydrazide **(13).*** A solution of **4** (1 mmol, 306 mg) in a mixture of AcOH acid and Ac_2_O (50 m, 1:1 *v*/*v*) was refluxed with stirring for 8 h. The reaction mixture was dropped onto iced-water. The obtained precipitate was filtered off, washed with water, and recrystallized from ethanol to give N-acetyl derivative **13**. Yield 70%, m.p. 235–237 °C. IR (KBr, cm^−1^): ν 3369-3232 (NH, NH), 1732 (C=O). ^1^H-NMR (DMSOd_6_) δ_H_: 1.86 (s, 3H, CH_3_), 4.68 (s, 2H, CH_2_), 7.10–7.70 (m, 4H, thiophene-H), 8.44 (s, 1H, pyrimidine-H), 10.70, 10.82 (2s, 2NH, disappeared with D_2_O). ^13^C-NMR (DMSO-d_6_) δc: 20.1 (CH_3_), 50.1 (CH_2_), 119.9, 122.0, 122.2, 126.8, 127.8, 128.7, 131.3, 136.4 (8C, thiophene-C), 157.0 (1C, pyrimidine-C), 162.8, 165.6, 169.1 (3C, 3C=O). Mass spectrum, *m*/*z* (EI, %): 348 (M^+^, 100), 349 (M^+^ + 1, 16). Analysis for C_14_H_12_N_4_O_3_S_2_ (348.40): Calculated: C, 48.27; H, 3.47; N, 16.08; S, 18.40. Found: C, 48.20; H, 3.40; N, 16.00; S, 18.32.

*Synthesis of compounds **14** and **15.*** To a mixture of **13** (1 mmol, 348 mg) and ethylcyanoacetate or malononitrile (1 mmol) in EtOH (40 mL), a few drops of triethylamine were refluxed for 8 h, poured into iced-water. The precipitate was filtered off, and crystallized from EtOH to obtain compounds 14 and **15**, respectively.

*N-(6-Amino-4-hydroxy-2-oxopyridin-1(2H)-yl)-2-(4-oxo-5-(thiophen-2-yl)thieno[2,3-d]-pyrimidin-3(4H)-yl)acetamide **(14).*** Yield 75%, m.p. 280–282 °C. IR (KBr, cm^−1^): ν 3492-3196 (OH, NH_2_, NH), 1420, 1680, 1653 (3 C=O). ^1^H-NMR (DMSO-d_6_) δ_H_: 4.15 (s, 2H, CH_2_), 4.95, 5.70 (2s, 2H, 2CH), 6.50 (s, 2H, NH_2,_ disappeared with D_2_O), 7.15–7.74 (m, 4H, thiophene-H), 8.50 (s, 1H, pyrimidine-H), 10.25 (s, 1H, OH, disappeared with D_2_O), 10.65 (s, 1H, NH, disappeared with D_2_O). ^13^C-NMR (DMSO-d_6_) δc: 49.00 (CH_2_), 116.1, 120.2, 121.8, 126.7, 127.9, 128.9, 131.3, 136.5 (8C, thiophene-C), 86.5, 100.2, 145.5, 158.7 (4C, pyridine-C), 156.2 (1C, pyrimidine-C), 164.1, 165.5, 169.5 (3C, 3CO). Mass spectrum, *m*/*z* (EI, %): 415 (M^+^, 75). Analysis for C_17_H_13_N_5_O_4_S_2_ (415.44): Calculated: C, 49.15; H, 3.15; N, 16.86; S, 15.43. Found: C, 49.05; H, 3.10; N, 16.80; S, 15.35.

*N-(2,4-Diaminopyridin-1(2H)-yl)-2-(4-oxo-5-(thiophen-2-yl)thieno[2,3-d]-pyrimidin-3(4H)-yl)acetamide **(15).*** Yield 75%, m.p. 290–292 °C, IR (KBr, cm^−1^). ν 3460-3345 (NH, NH2), 1680, 1653 (2C=O). ^1^H-NMR (DMSO-d_6_) δ_H_: 4.13 (s, 2H, CH_2_), 4.60 (s, 2H, NH_2,_ exchangeable with D_2_O), 5.60–6.10 (m, 4H, 4CH), 7.10–8.72 (m, 4H, thiophene-H), 8.47 (s, 1H, pyrimidine-H), 9.12 (s, 2H, NH_2,_ disappeared with D_2_O), 10.32 (s, 1H, NH, disappeared with D_2_O). ^13^C-NMR (DMSO-d_6_) δc: 48.00 (CH_2_), 115.1, 120.0, 121.8, 126.8, 127.7, 128.7, 131.3, 136.5 (8C, thiophene-C), 78.5, 105.1, 118.5, 139.7, 150.0 (5C, pyridine-C), 157.0 (1C, pyrimidine-C), 165.6, 169.3 (2C, 2CO). Mass spectrum, *m*/*z* (EI, %): 400 (M^+^, 50). Analysis for C_17_H_16_N_6_O_2_S_2_ (400.48): Calculated: C, 50.99; H, 4.03; N, 20.99; S, 16.01. Found: C, 50.90; H, 4.00; N, 20.90; S, 15.95.

### 3.2. Biological Evaluation

#### 3.2.1. Cytotoxic Assay

“Human breast cancer cells (MCF-7) and normal non-tumorigenic MCF-10A cells were used throughout the work. Cells were obtained from ATCC, Gaithersburg, MD, USA. Standard MTT assay was used to explore the possible cytotoxic effects of the synthesized compounds [41,42]. Medium composition, cultivation conditions and assay performance were exactly the same as our previous work [43,44]. Cells were treated with varying concentrations (0–1 µM) of the compounds prepared in DMSO. After MTT addition, the absorbance of the dissolved formazan crystals was read at 570 nm [45]. The IC_50_ values were obtained with linear regression equations using Origin^®^ 6.1 software (Origin Lab Corporation, Northampton, MA, USA)”.

#### 3.2.2. Human Breast Cancer Xenograft Animal Model

“In this work, MCF-7 mouse xenograft model was used. The animal protocol was approved by the Institutional Animal Use Ethics and Care Committee of the University of Alabama at Birmingham (50-01-05-08B). Female athymic pathogen-free nude mice (nu/nu, 4–6 weeks) were purchased from Frederick Cancer Research and Development Center (Frederick, MD, USA). To establish MCF-7 human breast cancer xenografts, each of the female nude mice was first implanted with a 60-day (subcutaneously, s.c.) slow release estrogen pellet (SE-121, 1.7 mg 17α-estradiol/pellet; Innovative Research of America, Sarasota, FL, USA). After 24 h, grown cells were harvested, washed twice with serum-free medium, resuspended, and injected subcutaneously (5 million cells/0.2 mL) into the left inguinal area of the mice. During the experiment, animals were checked periodically and the percentages of tumor growth, as well as animal weights, were recorded. Every 48 h, the size of the tumor was recorded by measuring two perpendicular diameters of the tumor and tumor volume was calculated according to Wang et al. [46]”.

“Treated animals and control groups (7–10 mice/group) received different compounds and vehicles, respectively. The tested compounds were dissolved in PEG400:ethanol:saline (57.1:14.3:28.6, *v*/*v*/*v*), and injected intraperitoneal (i.p.) at doses of 5 and 10 µM/kg/d, 3 d/wk for 3 weeks. The higher dose (10 µM/kg/d, 3 d/wk) inhibited MCF-7 xenograft tumor growth”.

#### 3.2.3. Pim-1 Kinase Inhibitory Activity

##### Materials and Methods

“The kinase inhibitory activity of the synthesized compounds was determined using the Kinexus compound profiling service, Canada. Compounds were tested at 50 nM concentration. The kinase used was cloned, expressed and purified using proprietary methods. Quality control testing is routinely performed to ensure compliance to acceptable standards. ^33^P-ATP was purchased from PerkinElmer. All other materials were of standard laboratory grade”.

##### Pim-1 Kinase Protein Assay 

“The protein kinase target profiling was executed via employing a radioisotope assay format. All the assays were performed in a prepared radioactive working area. The protein kinase profiling assays were performed at room temperature for 20–30 min in a final volume of 25 µL according to the reported method [47]”.

## 4. Conclusions

During the current work, different new **14** thiophenyl thienopyrimidinone derivatives were synthesized using variable cyclization and condensation routes. The synthesized derivatives showed promising potential biological potentials for their use in the pharmaceutical industry. They revealed higher in vitro cytotoxic activities against breast cancer cell line MCF-7 in comparison to known drugs, e.g., Cisplatin and Milaplatin. Furthermore, the prepared derivatives proved to be less toxic against the non-tumorigenic MCF-10A cell line. In vivo studies also showed potential reduction in tumor growth in animal models for all synthesized derivatives compared to control animals. Finally, mechanism of action studies showed that the newly synthesized derivatives exert their anticancer effects through the inhibition of pim-1 kinase enzymes.

## Figures and Tables

**Table 1:** The average weight of each group of mice treated with drug and the control group.

Drugs	Average Weight of Animals in Grams after Days											
	0	2	4	6	8	10	12	14	16	18	20	22
Control	28	28	28	28	28.2	28.	28.3	28.4	28.4	28.4	28.4	28.4
3	28	28	28	28	28.2	27.9	27.8	27.7	27.7	27.6	27.7	27.7
4	24.2	24.2	24.1	24.0	23.9	23.9	23.7	23.7	23.6	23.6	23.6	236.
5	22.9	22.9	22.9	22.8	22.8	22.7	22.8	22.7	22.9	22.6	22.6	22.5
6	23.7	23.7	23.6	23.6	23.5	23.5	23.5	23.4	23.4	23.5	23.5	23.5
7	27.3	27.3	27.2	27.2	27.2	27.1	27.1	27.1	27.0	27.0	27.0	27.0
8	25.3	25.3	25.2	25.2	25.2	25.1	25.1	24.8	24.7	24.6	24.5	24.2
9	24.1	24.1	24.0	24.0	24.0	24.0	23.7	23.7	23.6	23.6	23.6	23.6
10	24.9	24.9	24.9	24.9	24.8	24.8	24.8	24.4	24.4	24.4	24.4	24.4
11	26.6	26.6	26.6	26.5	26.6	26.6	26.4	26.3	26.3	26.3	26.3	263.
12	27.4	27.4	27.4	27.3	27.3	27.3	27.2	27.2	27.2	27.1	27.1	27.1
13	25.4	25.4	25.4	25.4	25.4	25.3	25.3	25.3	25.3	25.3	25.3	25.3
14	26.5	26.4	26.3	26.2	26.1	25.9	25.9	25.9	25.8	25.8	25.7	25.7
15	26.1	26.1	26.1	25.8	25.8	25.7	25.7	25.6	25.6	25.5	25.5	25.2
